# Address Introductory to the Twenty-First Annual Course of Lectures in Rush Medical College, Delivered Oct. 7th, 1863

**Published:** 1863-11

**Authors:** E. Ingals


### CHICAGO MEDICAL JOURNAL.

VOL. VI.] NOVEMBER, 1863. [No. 11.

### ORIGINAL COMMUNICATIONS.

### ADDRESS INTRODUCTORY

TO THE TWENTY-FIRST ANNUAL COURSE OF LECTURES IN RUSH MEDICAL COLLEGE,

*Delivered October 7th*, 1863,

By Professor E. INGALS.

*Gentlemen, Members of the Class :*

The exercises of the evening are initial to the twenty-first annual course of lectures in the Rush Medical College, and it is always our custom on like occasions to receive the class with a general introductory address, and in compliance with this custom, I am deputed by my associate members of the faculty to meet you to-night, and in their name I bid you a cordial welcome to these halls, dedicated as they are to the teaching and learning of the Science of Medicine.

We witness this evening with peculiar pleasure the presence of by far the largest class that has ever been convened at the commencement of any session within the walls of this College, and we accept it as a compliment from the profession at large, to whom we owe so much for their generous sympathy and support, and I desire in this public manner to express to them our thanks for this unmistakable evidence of their confidence and approval.

I regret the absence from our circle to-night of the cheerful face of one by whom it has before been adorned. Prof. Miller has been spending a few months beyond the ocean, in lands made interesting by their antiquity, visiting their hospitals, associating with members of the profession and observing their modes and means of teaching. He designed sailing from Liverpool on the 30th ult., and already his feet must well-nigh press again his native land, and in a few days we may expect him to resume his labors before the class, physically invigorated by the recreation of travel, his mind enriched by observation, and bringing with him something additional to our present means for illustrating the several branches of the course.

But my regret at the temporary absence of one, is more than compensated by the permanent addition of Dr. E. L. Holmes, another teacher, to our ranks. Of his modest worth, and rare scientific attainments in the branches in which he proposes to instruct you, his presence forbids me to speak in such terms as my feelings would prompt and truth would justify. I trust that our efforts to give you something more than ordinary opportunities to become familiar with diseases of the eye and ear, and their proper treatment, will meet your approbation and redound to your advantage.

During the current course of lectures our relation to each other is to be of the most interesting nature—the relation of teacher and pupil; and the Faculty will esteem themselves fortunate if their intercourse with the class should prove no less pleasant than it has been during former sessions. More than this we do not hope, and can scarcely desire. The same kindness of treatment from you, the same courteous conduct and proper demeanor, an equally sedulous attention to our teachings, will shed over our minds the invigorating dews of a perpetual joy, and go far to recompense us for all the toils incident to the place we occupy.. On our part, I may safely pledge that it shall be our earnest endeavor to merit no less than this by the rendition to each of you of every act of kindness that fortune may cast in our power, and by the diligent and faithful discharge of all the duties that we owe you. Amid the multitude of thoughts that are fit to be uttered on an occasion like this, it is difficult to decide which to choose or which to reject, but it shall be my endeavor to say something that may be *useful* to you, to aid you along the struggle of your student life, as well as in that more real life that shall follow, when at the bed side of the sick you are called upon to apply the knowledge you here acquire. For you will not begin too soon to view your future lives, as it were, in perspective, to indicate as well as you can the general course they shall take, and by the exercise of a judicious foresight assure to yourselves a pleasant and prosperous voyage, a valuable freight, and the safe attainment of a desirable haven. I presume I do not err in supposing that a large majority of your numbers are but commencing the study of medicine and fitting yourselves to enter upon the duties incident to its practice.

To such the future of your lives is as an unknown land, or an ideal world, illumined only by the rays of hope or painted by the exuberance of a youthful fancy. You do not know the difficulties of your present position; the obstacles that will interpose themselves across your several ways; the seductive influences that will environ you, ever inviting you to leave the straight and narrow path in which you now promise yourselves you will walk. Happy should I be, could I point out to you some of these difficulties and dangers, to the end that you may the better overcome or avoid them, thus rendering your course the more easy, your success the more assured, and your usefulness the more abundant. With these objects in view, my interest in your welfare prompts me to the usually thankless task of proffering you some words of advice and direction, and for this office my past experience has not been unfavorable, for at a day distant enough to correct the false colorings that so generally surround objects but little removed from our mental vision, and yet not remote enough to be obscured by frogetfulness, my place of observation was the same that you occupy to-night, and what is past to me, *has*

*been*, essentially, as the future *shall be*, to you, and as I look upon your faces they reflect upon me the retrospect of my own life, and memory with its wondrous powers brings back upon my mind each link in the brief chain of former years, and it seems but yesterday, since in fulfilling my purpose of attending my first course of medical lectures, I found myself the denizen of a busy, struggling city, so in contrast with all my previous tranquil experience, and I do not forget that the remembrance of home and the events of my boyhood would obtrude themselves upon my mind, despite the forbiddings of will. I remember too, the joyous feelings some of you experience, as I returned to my second course of lectures, and found myself surrounded by scenes made glad by memory of the pleasures of a former session, and the meeting again of college friends, and the cultivation of pleasant intimacies with those who were with us for the first time; for I count it as my good fortune that I entered the profession across the threshold you have just passed; that as a student, I occupied the seats you now occupy, and listened to the teachings of some by whom you shall profit, spared yet in the good providence of God to gladden us by their presence. Similar motives, I doubt not, have operated on most of your minds to induce you to make the study and practice of medicine the business of your lives, and to such of you as are fitted by natural endowments for this noble work, and who shall apply the future of your lives faithfully and conscienciously to its performance, I cannot but offer you my hearty congratulations on the selection of a vocation that you have made.

I know it is much the custom of medical men to speak differently on this subject, and to be always underrating the standing of the profession and the desirableness of a place in it; who tell us that our services are little appreciated by the public; that we have more troubles, weightier labors, greater responsibilities, and fewer rewards for all than any other class of society. Against these sweeping assertions, founded as I conceive them to be, mainly in error, I protest. They are conclusions drawn from distorted views of life, which multiply and magnify the evils that lie close around us, until they so completely overcast the mind's eye with their somber shadows that ills more remote and beyond the field of our own endeavors escape our observation and elude our knowledge.

> "'Tis distance lends enchantment to the view,
> And clothes the mountain with an azure hue."

I must congratulate you too, on the time during which fortune has ordained that you shall study and practice your profession. Never before has so propitious a season offered its encouragements to the youthful devotee of the study of medicine. The mass of medical knowledge garnered and transmitted from generation to generation has been constantly augmenting and in late years with an accelerated speed, and while new truths have been added, errors of the past, hallowed perhaps by the sanction of great and honored names, have been rigidly interrogated and submitted to the crucial test of unbiased observation, and falling before it, have been discarded; the pathology of some diseases, until recently unrecognized, has been made clear; the treatment of many maladies and injuries has been vastly improved; new remedies of marvelous power and efficacy, beneficient gifts from God to his suffering children, have been placed in your hands, and the proper uses of the old have become better understood. Of a truth it may be said, "Other men labored and ye are entering into their labors." Then too, the standing of the profession in the Republic was never so exalted as at present, or its essential necessity to the best interests of society so fully recognized. In the dull and long monotony of peace, men had come to receive the benefits that flow from the knowledge of Medical Science without appreciating fully their magnitude, or the source from whence they came; but the tread of martial hosts and the din of conflict soon awakened them from this listlessness. When vast armies were assembled people were not long ignorant or unmindful that it was disease and not the sword that was the great reaper in the human harvest of war, and when we witnessed our many battle fields, encumbered with the torn and shattered forms of those noble men who had given themselves with a prodigality, cheerfulness and devotion unparalled in the annals of nations as a "living sacrifice" for their country's good, our sympathies were aroused and we felt under a deep sense of obligation to extend to them every aid and solace in our power. Here was a real emergency in life; it was not the vapors of imagination or affectation; it was not the complainings ot the idle or luxuriant; it demanded something more than *adcaptandum* phrases, simple smiles, obsequious attentions, and sweet-scented nothings; here was a manly work to be done; it had to be squarely met; it would tolerate no trick or subterfuge; it was the trial by fire that should sift those who had knowledge and power from charlatans and pretenders. Where did the nation turn in this pressing exigency? Where could it but to the Mecca of true science? Who thought of committing to any of the fungi, the barnacles, the parasites, that shelter themselves beneath the shade of legitimate medicine, the safety of the nation, as bound up in the physical well-being of the soldiers whose stout hearts and strong arms bore aloft amid the smoke and carnage of battle the beloved banner of our fathers and of us, whose stars have illuminated every bright page in our nation's history? Few even of the dupes of these delusions. And it is a source of pride to members of the profession, and of congratulation and joy to every citizen, that the Medical Staff of the army acquitted itself so nobly in this trying and supremely responsible position.

For a time after the war broke out we were afflicted with a set of men who constituted themselves the critics of its conduct, who felt an overshadowing consciousness of their own capacity; men who had never looked upon a regiment of soldiers or stepped across the threshold of a hospital; who had never studied the art of war or the Science of Medicine. If the army moved, or was inactive, it alike received their censure. They usually represented our generals as besotted, or imbecile, or disloyal, and ever predicted that we should only meet disappointment and discomfiture until something of their wisdom should be infused into the conduct of public affairs. But it was upon the medical staff of the army that these persons most delighted to make disparaging comments, and they seemed never to tire of drawing on their imaginations for descriptions of the total unfitness and heartlessness of the army surgeons. But this has changed now; the war has taught us lessons of wisdom; the self-assurance of many is less arrogant; fragile bubbles have not withstood its shock. The deeds of those of the grand army whose mission it is to destroy, as exhibited on scores of well-fought fields, have stifled the stupid cavailings of these censorious meddlers, and the aggregated reports of the medical staff, whose merciful mission and labor it is to save life, and not to destroy, to assuage pain and not to inflict, have placed the Medical Department of the Army safe in the confidence and attachment of both army and public, and high above the levity, or malice, or ignorance of these trifling and ill-natured critics. It is not an exaggeration to say that no army has been more ably and faithfully served by its medical guardians than ours has been, and its marvelous efficiency has been due, in no inconsiderable degree to this cause. Reflecting upon this, I cannot repress emotions of pleasure when I consider the part the alumni of the Rush Medical College have sustained in this work. From all over the Northwest they have pressed into the service with patriotic fervor, and their record in camp and field has filled us, their former teachers, with pride and made us their debtors. All honor, I say, to every surgeon who has taken upon himself the dangers, the labors and sacrifices incident to this holy work; and you may remember that something of this glory will be reflected along your pathways, and it is more than likely, too, that in other wars, if not in this, you may be called upon to sustain in the field the reputation acquired by the army surgeons now in service; for his faith must be both strong and blind, that shall look in this generation for the promised period "when swords shall be beaten into plow-shares and spears into pruning hooks." The present too, is a favorable time for entering the profession on account of the increased demand for medical men that has been induced by the necessities of the war. Great numbers of physicians, long established in extensive and lucrative business, have left all at their country's call, and vacancies thus open invite your presence.

It behooves every one commencing the study of medicine to consider carefully whether he is fitted by nature for its successful prosecution, and if he is not able to answer this question affirmatively to his own mind, then it is the part of wisdom and duty to abandon at once all thought of attempting so difficult an undertaking. The *highest excellence* in the profession demands the richest endowments with which nature in her most prodigal moments clothes the human mind. The apprehension must be ready; the reasoning powers good; the judgment correct and rapid; the memory tenaceous; and that all these may be quickened into vitality, the mind must be so constituted as to *love labor;* there must be a steadfastness of purpose that pursues its object in the face of every obstacle, with unflagging energy and unremitting zeal; and all these endowments should be permeated and cemented by a congenial nature, and the breast should be strong with chords attuned in harmony with the vibrations of hope and joy, of apprehension and sadness, that in all times and in all circumstances emanate from chambers where sickness and death hold sway. Without probity and truth; a character above the petty and trivial vices too commonly inherring in human nature; a nice sense of personal honor, which is at once the brightest ornament and the most secure protection of the mind by which it is shielded and adorned, there is no hope for a really exalted position in the profession of medicine. Without these, it is true, some may *appear* to be successful, but it is a *seeming* and *ficticious* success, not *real;* their gilding is exterior, and the interior of their characters is neither to be admired nor commended. They are "like unto whited sepulchres, which indeed appear beautiful outward, but are within full of dead men's bones and of all uncleanness." If, after a careful self-examination, you are satisfied that nature has given you qualifications sufficient to enable you to take an honorable place in the profession, and having "put your hand to the plow," do not look back, nor suffer yourselves to be swerved from the purpose of your sober judgment by the labors that surround, the obstacles that oppose, or the vexations that annoy you. The aspirations of the morning of life may be suffiicently elevated, the objects desired and sought may be worthy, and the resolution to persevere unto the end may seem well fixed, but as we go forward to the accomplishment of our purpose, unlooked-for opposing forces are found in our way, annoyances we did not anticipate are thrust upon us, cares vexations and burdens, as incidental to the full sunshine of prosperity as the shadow is to the substance, are multiplied or magnified, distant hope is obscured by closely investing clouds, and with a faint heart we retire ingloriously from the field before the battle is well begun. You should always avoid being influenced unduly by what is near and transient, and to govern your actions by the immutable truths, discernable enough, but which may lie beyond the range of easiest vision.

Remember that obstacles if attacked with vigor, judgment, and persevernce, are generally less than they seem, and that "lions in the way," are oftener imaginary than real. While then you devote yourselves manfully to the allotted task before you, you should be inspired by a single purpose, and that the acquisition of medical knowledge. A jealous goddess presides over our time-honored science, who grants favors to no suppliant who brings to the shrine divided affections, and we scarcely need the wisdom of inspiration to know that "no man can serve two masters." An honorable place in the profession will demand of you a lifetime of labor, both mental and physical, for such is the inevitable price for which the reward is bestowed, and if you suppose it may be attained through a life of easy indolence, the sooner you are undeceived the better; and if you are not determined resolutely to assume its labors, you had best seek some easier occupation, as another profession, some of the mechanic arts, trade, commerce or agriculture.

Consider what will be demanded of you—the varied knowledge that will add to the intelligence and success with which you will treat disease. The disciple of medicine must know both the physiology and pathology of nature—he must trace as well as he can the causes that assure health or occasion disease—he must learn the action of matter upon matter within the human economy, and this both under the influences of the vital forces and pathological action—anatomy should be so familiar to him, that he shall know the position and relative location of nerves and vessels as he does the streets and walks of his native village, and of important organs as the different apartments of his own dwelling—remedial agents must be closely studied, their action observed and the circumstances, time, and manner for their proper employment well understood—each disease is to be so carefully investigated as to be readily and surely recognized, its true pathology must be known, and its proper treatment comprehended—the wonders of chemistry must stand revealed to the mind. No slothful life will fit one for the delicate and trying duties of the obstetric practitioner, while the higher and broader field of surgery will demand all of labor and capacity. As you come day by day to realize the magnitude of the labors involved in what I have stated, you will not at all times find yourselves above feelings of discouragement and despondency, but the sunlight of manly endeavor will dispel the clouds and shed in upon your minds rays of genial warmth and vivifying power.

Remember how much may be compassed by determined energy, patience, and industry. In the material world, man finds the high-topped hill placed by an Omnipotent hand across the pathway of commerce, and the timid and irresolute turn from it and say it is insuperable; but sturdy, intelligent labor approaches it and smites it with steel, and little by little it crumbles beneath the steady stroke, until the light of day shines through where darkness was, and the ponderous train goes smoothly on its easy way beneath the mountain's base. So the accumulated obstacles that lie before you in the profession, seeming almost insurmountable, will in a manner that shall astonish you, succumb to patient, persistent, well-directed toil. Your collegiate course will be but an epitome of your whole professional life, for you can never properly practice medicine after you cease to be students, though your studies now and after you have entered on practice will differ somewhat, both in their scope and nature, yet they will either be but segments of the great circle, and one will be complemental to the other. I would impress upon you as of paramount importance, that in your pupilage you master the rudiments of the knowledge of your profession in the most thorough manner, for in these are its sure foundations laid. We live in a city that for rapidity of growth is the marvel of history, and the exigencies incident thereto have sometimes compelled us to extemporize what is usually done at greater leisure and in a more permanent manner. In this spirit we occasionally see tenements erected, not on foundations wrought from the stable rock, but placed only on pine sticks set upright in the earth, and ere a house properly constructed is settled well upon its base, these topple to their fall. The same holds true in the effort to erect a professional fabric. If the foundation is not firmly laid in a thorough mastery of the elements of the science, the whole superstructure will prove faulty and unstable. A proper foundation may be put down, and yet the building may not be completed from a lack of steady purpose to carry it forward, but if the foundation itself is neglected, no subsequent diligence will be likely to atone for this first omission. As to the best plan for conducting your studies while in attendance upon lectures, I can suggest to you no unvarying rules In this, as in all things to a great extent, each must be a law u› himself. While here, you will acquire knowledge most rapidly from the lectures to which you will listen, and to the understanding and retaining of these, you should give all your efforts. Some will accomplish this best by taking brief notes in the lecture room ; others by trusting to the memory, at least until they have reached their rooms, when they may fix what they have heard better on the mind by a manuscript of the more important facts. Points that are still obscure should be elucidated by reference to the standard text books. There will be some in every class who should give a constant spur to energies that a natural indolence of character may always tend to lull into inactivity and repose, and yet on this point my observation of classes of medical students leads me to inculcate upon you the necessity of diligent and hard study, with some degree of reserve, for it is not unfrequent that the ardor of youthful hope and generous endeavor, require to be repressed within narrower limits rather than to be stimulated to greater exertions. It is by no spasmodic efforts of overtasked powers that you are to acquire knowledge or to achieve success, but rather by such as are easy and long sustained. If you would accomplish most during the lecture term, look well to your health, and do not place too great a weight on your physical powers. Your attendance on lectures and your anatomical studies will be very laborious, and to fit yourselves properly to meet this effort you should be careful to take your regular rest and a full measure of it. Late hours are pernicious to health, and consequently to the rapid scientific progress of students. Do not be misled by the long fashionable talk about the "midnight oil," for adequate rest is better for you than unseasonable midnight oil, and has been indulged to the full requirements of nature by those in all time who have made the most of life, and who have left enduring footprints upon the earth in their beneficent works. You will profit little by sleepy vigils over your text books, and you will unfit yourselves for your duties in the lecture room, where you should bring a clear head and active faculties. Take proper exercise in the open air, and nourish and support your bodies well.

Melancholy memories of the last session have led me in this train of thought and impressed these convictions more deeply on my mind. We then saw one of the most promising of our number cut down untimely from among us, in the very spring-time of a hopeful life, by a disease aggravated, if not induced by a too laborious confinement to his studies. 'Twas "Science' self destroyed her favorite son." Do not however from what I have said, take license to squander your time in idleness, or dissipate it in pleasure, when neither rest nor recreation is required.

You will pardon me if I point your attention to a danger that may be as obvious to you as to me, which has been the frost that has blighted numbers of the most promising buds of hope the profession has ever nourished, whose early hopes have ended only in disappointment and sadness. I allude to the practice of insobriety. Against its malign and seductive influences you cannot pledge yourselves too strongly, or be too vigilant in keeping beyond the reach of danger, and this will never be more imminent than when released from the restraints of home, and exposed to the allurements of a city and the convivialities of college life, and you are fortunate if any who cannot recall to your minds mournful examples of the blight and ruin that follow in the train of the practice of this vice. You may be reflecting that as yet I have promised you nothing as incidental to your professional career but labor, anxiety and vexation—that its responsibilities will not be less than appertain to any private station, inasmuch as you are to hold the health and life of your patrons in your hands, subject to the skill and knowledge you shall possess, and influence in no small degree both their finite and infinite interests—and you may be ready to ask what shall repay for all this? To this I would answer, the rewards are as adequate and certain as are found in any of the pursuits to which man lends his efforts. I would not have those just starting off on the active duties of life, whether as physicians or in any other walk, to expect too much. Inordinate and unreasonable hopes, implanted in the minds of multitudes of youth, have exerted upon them only a baneful influence, serving to cover them with disappointment and fill them with dissatisfaction at their lot and station. The young man just entering some college or academy, the better to perfect his education, is told by his partial parent, and over-zealous teacher that there is no station he may not hope to fill—that the Presidency of the Republic—which they usually represent as the most desirable of all sublunary things—does not lie beyond the bounds of his reasonable ambition, and all this is confirmed by the citation of servant boys and rail-splitters who have achieved this elevated position. He is never permitted to forget that every man is the "architect of his own fortune," and "*can be* what he *wills to be*," and he is always instructed to "aim at the sun," not it is true in any hope of hitting that luminary, but that he may reach higher than if his aim is some more humble object. This is not the custom of the best marksmen, they do not calculate all the nice chances of the loss of momentum, but ever hold level on the target, and if it is beyond the range of their piece they provide themselves with one of greater capacity, or go nearer. I would have you apprehend what lies within the compass of your reasonable hopes—what you have a right to expect—that you may put forth suitable efforts to attain this, and securing it, therewith to be content. You every one expect to reap success in your profession, else you would not assume it—but what is it to be successful? You cannot *all* reasonably expect, the *most eminent* success. You may seek it, and approach it as nearly as may, but do not be disappointed if you do not attain it, or conclude that therefore your lives have been failures.

There is only room in society for a few *great* men, as there is only room in the forest for a few trees of the most ample girth and towering and wide-spread branches. Numberless oaks, springing at the same time from a generous soil all promise equally well—but one will overshadow and dwarf the rest, and no wisdom can divine in the early race which shall be the giant and which the pigmy. Nor is the most exalted position necessarily the most desirable. It is the tall oak that first feels the lightning and the blast, while the humble and sheltered are comparatively secure. The same holds true in the social race and amid ambitious strife.

> "He who ascends to mountain-tops shall find
> The loftiest peaks most wrapt in clouds and snow;
> He who surpasses or subdues mankind,
> Must look down on the hate of those below.
> Though high above the sun of glory glow,
> And far beneath the earth and ocean spread,
> Round him are icy rocks, and loudly blow
> Contending tempests on his naked head,
> And thus reward the toils which to those summits led."

What you have a right to expect from your professional labors, and what you may nearly all attain, is this—and if you reach it endeavor to be satisfied therewith, and do not embitter your lives by sighing for positions that you see others occupy, which may be less felicitous than you suppose them to be. If you practice your profession conscientiously and intelligently, you will have the satisfaction of knowing that you have done something to mitigate the ills that afflict the race, and when your labors are over, you will look back upon a world made better and brighter by your sojourn in it. A place in the profession will enable you to make your social position one greatly to be desired. You may each of you be trusted, respected and beloved in the community in which you live, and into a large number of households may you come, on terms of closer intimacy and friendship than is permitted to any other person outside of the family circle. The privilege of ministering to the afflicted and relieving their distress is necessarily involved in the physician's duties, and sentiments of esteem and attachment cannot be otherwise than engendered thereby.

The practice of medicine not only gives suitable time for mental culture, but if properly pursued, *absolutely compels* it, while its duties intersperse an agreeable and salutary amount of physical effort. If opportunities are improved by the physician no man should possess more general information than he, and this in itself is a source of ever-present pleasure. To no other class are the secret workings of the human mind so constantly displayed, for before the physician it daily stands, absolutely unmasked. A place in the brotherhood of medicine will enable you at all times to command for yourselves and families the best professional care in sickness, a matter of no small moment. They will be safe in your own hands, or if you are obliged to assign them to the treatment of others, you will be able to form an intelligent judgment as to who is most competent to the trust. The practice of medicine will bring you a desirable pecuniary reward. It is true you will not be likely to amass great wealth by it, nor will you in any pursuit in which you may be so certain to arrive at a competency. The few who acquire large fortunes usually do so in those employments in which failures are numerous, and where great pecuniary risks are incurred. I know this is an object that is not usually held out to medical students, for they are generally taught that the duties of benevolence pertain above all others to the profession of medicine. I cannot see any well-grounded reason for this sentiment. That its members render in charity, of time and labor a greater proportion than any other class of society is true, and this because they are brought much in contact with their fellows in conditions of great suffering and want, and the patient, uncomplaining manner in which they minister to the necessities of such, without any hope of reward, save the silent commendation the conscience brings, reflects upon the profession its highest honor. The sum of gratuitous service thus cheerfully performed seems little appreciated by the public, and indeed it can only be realized by those by whom it is rendered. This benevolence is worthily bestowed, and is rendered, comparatively easily by the medical practitioner, for those who assume this profession are usually men of kindly feelings, and their daily association with the afflicted, and their efforts to abate their sufferings, quickens their sensibilities, and warms their sympathies, instead of chilling and dulling them as many erroneously suppose. I would not curtail, I would not repress the honorable impulses of the profession in this direction, I would rather encourage and promote them. But the great body of society lies beyond the limits which demand your charity, and from these you have a right to ask, and reason to expect, an ample pecuniary remuneration for the labors you will perform and the responsibilities you will bear. I would not enervate the profession, by taking from it the stimulus of the hope of reward, in which rests the vitality of every enterprise. In the pursuit of these objects do not be discouraged by difficulties —for it is conflict with these which attests and develops true manhood. It was the bearing of the army after a bloody and disastrous repulse from an assault on the works around Vicksburg, which extorted from its commander the highest commendation. Alluding to this, Gen. Grant, in his admirable report to his superiors in command, of the events that culminated in the capture of this city, says: "The assault of this day proved the quality of the soldiers of this army. Without entire success and with heavy loss, there was no murmuring or complaining, no falling back nor other evidences of demoralization." This is the spirit through which ultimate success is most surely attained. Though a single assault should prove unsuccessful do not, therefore, fall back or murmur or become demoralized. Do not expect to accomplish all this too soon. Reflect that it is the adequate work of a lifetime, and because you do not compass it in one year, or five, or ten, do not therefore lose heart, and trifle away your lives in futile efforts to achieve it by some dishonorable expedient as many have done. If prosperity attends you, though it be slow, pursue it with diligence, and remember that the race is not to the swift so often as to the untiring.

What I wish for you is, that from this point you take a comprehensive view of life, and so design it, and frame it, and build it, that it may become a stable, harmonious, perfect whole.

Make the foundations of your professional knowledge sure by mastering in your student life, the elements of the science; keep your moral characters unspotted; imbue yourselves with the spirit of true gentlemen; cultivate a sense of honor that will permit you to do no unworthy act; fix and adhere to the resolution to make your lives those of steady diligence; for by these means only will you become an ornament to your profession and a benefactor to your race.

